# Low compliance and need for changes in national isolation guidelines for group 1 nationally notifiable communicable diseases in Korea

**DOI:** 10.1186/1753-6561-5-S6-P324

**Published:** 2011-06-29

**Authors:** ES Kim, HB Kim, K-H Song, HS Lim, J Gwack, YH Choi

**Affiliations:** 1 Department of Internal Medicine, Seoul National University Bundang Hospital, Seongnam, Korea, Republic Of; 2 Department of Preventive Medicine, Dongguk University College of Medicine, Gyeongju, Korea, Republic Of; 3 Division of Epidemic Intelligence Service, Korea Centers for Disease Control and Prevention, Osong, Korea, Republic Of

## Introduction / objectives

The national guidelines for group 1 nationally notifiable communicable diseases(cholera, typhoid fever, paratyphoid fever, shigellosis, and enterohemorrhagic *Escherichia coli* (EHEC) infection) recommend a universal isolation of the patients and screening for fecal shedding after finishing antibiotic therapy in Korea. This study was performed to evaluate the adequacy of the isolation guidelines.

## Methods

We compared Korean guidelines with those of other countries. We also evaluated clinical and microbiological characteristics for confirmed cases and compliance with the guidelines in 20 Korean hospitals nationwide from 2000 to 2010.

## Results

Isolation and screening for fecal shedding was selectively applied according to type of disease, patient status, and their occupation in foreign guidelines. Among 535 confirmed cases (15 cases of cholera, 232 typhoid fever, 81 paratyphoid fever, 175 shigellosis, and 32 EHEC infections), only 3.7-26.7% of each disease were strictly compliant with the guidelines in Korea. Prolonged fecal shedding ≥7 days and secondary attack was more frequent in shigellosis and EHEC infection than in other diseases.

## Conclusion

This study shows that the present isolation guidelines were not realistic in Korea. Therefore, more detailed guidelines are necessary in Korea, which can be selectively applied for highly communicable diseases such as shigellosis and EHEC infections and for patient groups with high risk of secondary attack.

## Disclosure of interest

None declared.

